# Prognostic Value of Brain and Acute Leukemia Cytoplasmic Gene Expression in Egyptian Children with Acute Myeloid Leukemia

**DOI:** 10.4084/MJHID.2015.033

**Published:** 2015-04-20

**Authors:** Adel A. Hagag, Amal Ezzat Abd El-Lateef

**Affiliations:** 1 Department of Pediatrics, Faculty of Medicine, Tanta University, Egypt; 2 Department of Clinical Pathology, Faculty of Medicine, Tanta University, Egypt

## Abstract

**Background:**

Acute myeloid leukemia (AML) accounts for 25%–35% of acute leukemia in children. BAALC gene (Brain and Acute Leukemia Cytoplasmic gene) is a recently identified gene on chromosome 8q22.3 that has prognostic significance in AML. The aim of this work was to study the impact of BAALC gene expression on prognosis of AML in Egyptian children.

**Patients and methods:**

This study was conducted on 40 Egyptian children with newly diagnosed AML who were subjected to full history taking, clinical examination and laboratory investigations including: complete blood count, LDH, bone marrow aspiration, cytochemistry, immunophenotyping and assessment of BAALC Gene by real time PCR in bone marrow aspirate mononuclear cells before the start of chemotherapy.

**Results:**

Positive BAALC gene expression was found in 24 cases (60%) and negative expression in 16 cases (40%). Positive BAALC gene expression group includes 14 males and 10 females with mean age at presentation of 8.35±2.63 while negative BAALC gene expression includes 10 males and 6 females with mean age at presentation of 7.74±3.23 with no statistically significant differences between patients with positive and negative BAALC gene expression regarding age, sex and clinical presentations at time of diagnosis including pallor, purpura, splenomegaly, hepatomegaly and lymphadenopathy and laboratory investigations including WBCs and platelets counts, hemoglobin and LDH levels, and peripheral blood and bone marrow blast cell counts. There was significant association between positive BAALC gene expression and M1 and M2 compared with negative BAALC gene expression which is significantly associated with M4. There were statistically significant differences in disease outcome between positive and negative BAALC gene expression groups with higher rate of relapse and death and lower rate of complete remission and disease free survival in positive BAALC gene expression group compared with negative BAALC gene expression group. (p = 0.017).

**Conclusion and Recommendation:**

BAALC expression is an important bad prognostic factor in AML patients with normal karyotype and therefore we recommend its incorporation into novel risk-adapted therapeutic strategies to improve the currently disappointing cure rate of patients with AML.

## Introduction

Acute myeloid leukemia (AML) is a clonal malignant disease of the bone marrow in which hematopoietic progenitor cells are arrested at an early stage of development due to acquired genetic alterations that lead to failure of differentiation and to over proliferation.^1^ AML accounts for 25%–35% of the acute leukemia in children.^2^

Over the past decades, remarkable progress has been made in the treatment and the understanding of the molecular pathogenesis of acute myeloid leukemia. At present, up to 65% of pediatric AML patients experience long-term survival, owing to a more effective use of anti-leukaemic therapy, improvements in supportive care and better risk stratification ^(1)^.

It is important to identify prognostic markers that predict patient's outcome more precisely, thereby allowing the development of molecular risk-adapted treatment strategies that may improve the clinical outcome. By the use of molecular genetics techniques, such as reverse transcriptase polymerase chain reaction (RT-PCR), global gene expression profiling and/or direct sequencing, several recurring molecular alterations of prognostic significance have been identified in patients with cytogenetically normal AML (CN-AML).^3^

BAALC gene (Brain and Acute Leukemia Cytoplasmic gene), is located on chromosome 8q22.3.^4^ Expression of BAALC was found mainly in neuroectoderm-derived tissues and hematopoietic precursor cells. In hematopoietic cells*,* BAALC expression was restricted to the compartment of progenitor cells, whereas no expression was detected in mature bone marrow or circulating white blood cells.^5^

High BAALC gene expression was found in a subset of patients with AML, acute lymphoblastic leukemia (ALL), and blast phase of chronic myeloid leukemia (CML), whereas no BAALC expression could be detected in patients with chronic-phase CML or chronic lymphocytic leukemia (CLL) ^(4)^. Additionally, high BAALC gene expression occurs in glioblastoma, melanoma, and childhood gastrointestinal stroma tumors, suggesting an oncogenic role for BAALC gene. However, the mechanisms underlying the deregulated expression are unknown.^6^

The prognostic significance of BAALC gene was first shown in CN-AML with association with significant higher refractoriness to induction treatment, lower rates of complete remission (CR), poor overall survival (OS) and disease free survival (DFS) for patients with high BAALC expression independent of other prognostic molecular markers with a gene expression signature consistent with less differentiated AML blasts.^6^–^8^

## Aim of the Work

The aim of this work was to evaluate the prognostic value of BAALC gene expression in Egyptian children with acute myeloid leukemia.

## Subjects and Method

Approval for this study was granted by the ethical committee of Tanta University research center and written consent was obtained from the parents of all children involved in this study. The study participants included 40 Egyptian children with newly diagnosed AML being followed up under the Oncology Unit of the Pediatric Department in the period from March 2012 to December 2014 including 24 males and 16 females with their ages ranging from 3–16 years with a mean age value of 9.8±5.8 years. All patients were subjected to follow up for 2 years to evaluate their prognosis*.*

### Inclusion criteria

Children with cytogenetically normal AML.

All patients were subjected to the following:

❖ Full history taking.
❖ Thorough clinical examination with special attention to fever, pallor, purpura, hepatomegaly, splenomegaly, and lymphadenopathy.
❖ Laboratory investigations

### Specimen collection and handling

Three ml venous blood were collected under complete aseptic technique. They were delivered into 2 tubes: 1 ml blood into a tube containing EDTA for complete blood count and 2 ml blood into the plain tube for assessment of Lactate dehydrogenase levels. Two ml of bone marrow aspirate were drawn into a sterile tube containing EDTA for mononuclear cell separation for polymerase chain reaction (PCR).

Laboratory investigations include the following:

♦ Complete blood count.
♦ Lactate dehydrogenase (LDH)
♦ Bone marrow aspiration with cytochemical examination and immunophenotyping.
♦ Cytogenetic analysis
  Cytogenetic analyzes of bone marrow or peripheral blood were performed. Metaphase chromosomes were banded by G-banding technique and Karyotyped according to the International System for Human Cytogenetic Nomenclature. A minimum of 20 metaphases was required to be examined for any patient to be classified as having a normal cytogenetic study ^(9)^.
♦ Assessment of BAALC Gene by real-time PCR.
  Mononuclear cells were separated from the samples by centrifugation on Density gradient medium ^(10)^. RNA was isolated using an RNA easy Mini Kit, and the concentration of extracted RNA was evaluated by spectrophotometry (SPEC) ^(11)^. DNA amplification was done by real-time PCR using Gene Amp 5700 Sequence Detection System. Real-time PCR was used for the detection of BAALC gene ^(12)^.

BAALC mRNA expression was normalized simultaneously analyzing the glucose phosphate isomerase (GPI) gene. The relative BAALC expression was determined using the comparative cycle threshold method. BAALC were amplified using 1 μL cDNA, 1× master mix (IQ Mix; BioRad, Munich, Germany).

- Glucose phosphate isomerase (GPI) forward primer 5′-TCTTCGATGCCAACAAGGAC-3
- Glucose phosphate isomerase (GPI) reverse primer 5′-GCATCACGTCCTCCGTCAC-3
- Glucose phosphate isomerase (GPI) probe -′HEX-TTCAGCTTGACCCTCAACACCAAC-TAMRA-3′5
- BAALC Gene forward primer: 5′-GCCCTCTGACCCAAACAG-3′;
- BAALC Gene reverses primer: 5′-CTTTTGCAGGCATTCTCTTAGCA-3′;
- BALCC Gene probe: 5′-FAMCTCTTTTAGCCTCTGTGGT-3′;

Reactions were performed using real-time PCR 7000 sequence detection system (Applied Biosystems, Foster City, CA, USA) ^(12)^. A value of 0.166 for BAALC gene was attributed as cut off value, so BAALC gene expression was considered positive if BAALC gene expression is above 0.166. On the contrary, BAALC gene expression was considered negative if BAALC gene expression is below 0.166. Follow up of patients was carried out clinically and by blast count in BM on day 28 after two courses of induction therapy according to AML-protocol (MRC10). ^(13, 14)^.

#### Course 1 (ADE)

Aracytin 100 mg/m^2^ IV bolus every 12 hours on days 1–10 (20 doses), Daunorubicin 50 mg/m^2^ IV on days 1, 3, 5, Etoposide 100 mg/m^2^ (1 hour IV infusion) on days 1–5 and age adjusted intrathecal Aracytin at time of diagnostic lumber puncture.

#### Course 2 (ADE)

Aracytin 100 mg/m^2^ IV bolus every 12 hours on days 1–8 (16 doses), Daunorubicin 50 mg/m^2^ IV daily on days 1, 3, 5, Etoposide 100 mg/m^2^ daily (1 hour IV infusion) on days 1–5 and age-adjusted intrathecal Aracytin on day 1 of course 2.

#### Course 3 (ACE )

Aracytin 1gm/m^2^/dose IV every 12 hours on days 1–5 (10 doses) and Etoposide 150 mg/m^2^/dose daily (1 hour IV infusion) on days 1–5 with age-adjusted intrathecal Aracytin on day 1 of course 3.

#### Course 4 (MIDAC)

Mitoxantrone 10 mg/m^2^ IV daily (short infusion) days 1–5, Aracytin 1gm/m^2^/12hours (2 hours IV infusion) on days 1–3 (6 doses) and age-adjusted intrathecal Aracytin on day 1 of course 4.

#### Course 5 (CLASP)

Aracytin 3gm/m^2^ IV every 12 hours on days 1, 2, 8 and 9 (8 doses), L-asparaginase 6,000 IU/m^2^ IM on days 2 and 9 (3 hours after completion of Aracytin).

Age-adjusted intrathecal chemotherapy with Aracytin: 20 mg for age less than 1 year; 30 mg for age of 1–2 years; 50 mg for age of 2 – 3 years and 60 mg for age of 3 year or older.

## Statistical Analysis

The patient’s data were collected and statistically analyzed using SPSS software statistical computer package version 12. All Data were expressed as in terms of mean values ± SD. The difference between two means was statistically analyzed using the student (t) test. A chi-square test (X^2^) and Fischer exact test was used as a test of significance. The log-rank test was used to assess survival. Significance was adopted at p < 0.05.

## Results

Positive BAALC gene expression was found in 24 cases (60%) and negative expression in 16 cases (40%).

Positive BAALC gene expression group (n=24) includes 14 males and ten females with a mean age at presentation of 8.35±2.63. Pallor was found in 20 cases, purpura in 22 cases, splenomegaly in 19 cases, hepatomegaly in 16 cases and lymphadenopathy in 8 cases. The negative BAALC gene expression group (n=16) includes ten males and six females with a mean age at presentation of 7.74±3.23. In this group pallor was found in 13 cases, purpura in 14 cases, splenomegaly in 13 cases, hepatomegaly in 11 cases and lymphadenopathy in 6 cases. There were no statistically significant differences between patients with positive and negative BAALC gene expression regarding age, sex and clinical presentations at the time of diagnosis. (Table 1).

There were no significant differences between positive and negative BAALC gene expression groups regarding WBCs and platelets counts, hemoglobin, and LDH levels, and peripheral blood and bone marrow blast cell counts. (The mean WBCs count was 75.51±51.31 in the positive BAALC gene group versus and 76.44±46.67 in the negative BAALC gene group, with a p-value of 0.845. The mean platelets count was 69.86±33.15 in the positive BAALC gene expression group versus 77.13±48.07 in the negative BAALC gene group, with a p-value of 0.712. The mean hemoglobin level was 8.13±1.57 in the positive BAALC gene group versus 7.86±2.37 in the negative BAALC gene group, with a p-value of 0.632. The mean LDH level was 1312.73± 695.63 in the positive BAALC gene group versus 1299.57±315.47 in the negative BAALC gene expression group, with a p-value of 0.082. The mean peripheral blood blast cells in the positive BAALC gene expression group was 63 ±20.05 versus 57.35±28.48 in the negative BAALC gene expression group, with a p-value of 0.416. The mean bone marrow blast cells in the positive BAALC gene expression group was 78.21±9.83 versus 75.39±21.79 in the negative BAALC gene expression group with p-value of 0.096) (Table 1).

There was a significant association between positive BAALC gene expression and M1 and M2 subtypes compared with negative BAALC gene expression significantly associated with the M4 subtype. In fact, of 24 cases with positive BAALC gene expression; 9 were M1, 13 were M2, and 3 M4 whereas of 16 patients with negative BAALC gene expression; 2 were M1, 4 M2, and 9 M4. The presence of positive and negative BAALC were statistically different in the FAB subtypes) (Table 1).

**Figure 1:**
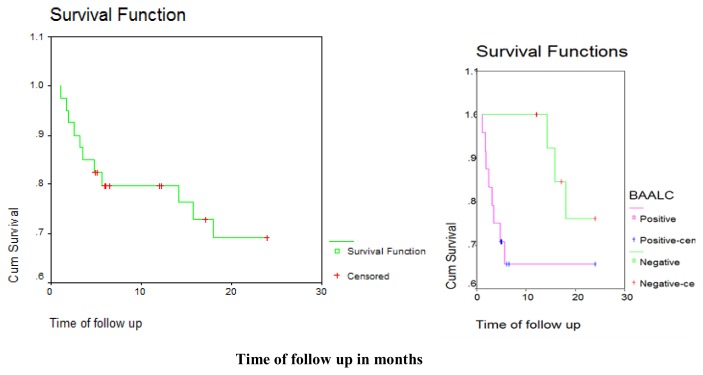
Kaplan Meir curve showing overall survival (OAS) in positive and negative BAALC gene expression groups.

**Figure 2:**
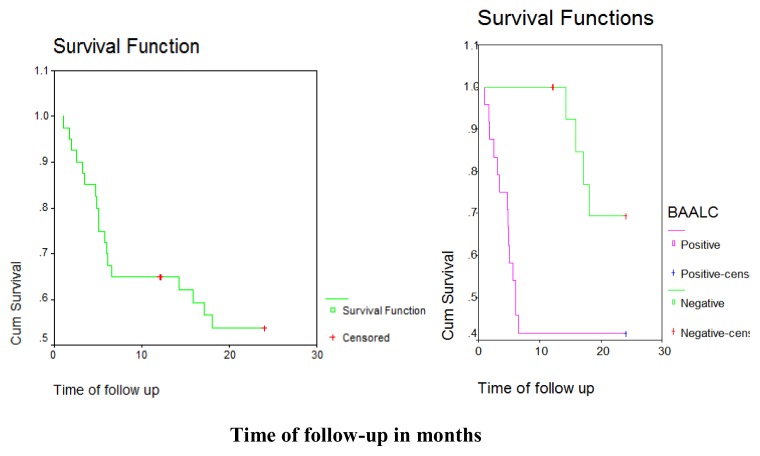
Kaplan Meir curve showing Disease Free Survival (DFS) in positive and negative BAALC gene expression groups.

There was a statistically significant difference in disease outcome between positive and negative BAALC gene expression groups with higher rate of relapse and death and lower rate of complete remission and disease free survival in positive BAALC gene expression group compared with negative BAALC gene expression group. (p = 0.017). Of 24 patients positive; 10 achieved complete remission, 8 died and 6 suffered from relapse, while of 16 patients negative; 12 achieved complete remission, 1 suffered from relapse and 3 died with a median disease-free survival in the positive group of 6.03 months compared with 21.63 months in negative group. (Table 2, 3 and Figure 1, 2).

## Discussion

Acute myeloid leukemia is a clonal malignant disease of hematopoietic tissue that is characterized by the proliferation of abnormal myeloblast cells principally in marrow and impaired production of normal blood cells ^(15)^. The prognosis of AML varies dramatically and is strongly influenced by a number of factors, including age, performance status, and cytogenetic and/or molecular alterations.^16^

In recent years, a major focus of molecular cancer research has been the analysis of genes that may be the cause of carcinogenesis (oncogenes).^17^ Multiple chromosomal and gene rearrangements have been identified in AML, such as MLL, PML/RARA, DEK/CAN, and AML1/ETO^18^ Chromosomal rearrangements involving the MLL gene at band 11q23 are the most common genetic alteration encountered in infant acute myeloid leukemia. Reciprocal translocation represents the most frequent form of MLL rearrangement.^19^ NRAS mutation varies considerabl**y** in patients with childhood AML and was found in about 15% of pediatric AML patients in some studies.^17^, ^20^

The present study was designed to use Real-time PCR analysis to study the prognostic value of BAALC gene expression in Egyptian children with AML.

In this study, positive BAALC gene expression was found in 24 cases (60%). This is in substantial accord with Yahya et al 2013,^21^ who found high BAALC gene expression in 22 of 45 patients (48.9%) and Damiani et al 2013,^22^ who found BAALC gene overexpression in 87/175 (50%) of their studied patients.

In the present study, there were no statistically significant differences between patients with positive and negative BAALC gene expression regarding age, sex and clinical presentation at time of diagnosis including pallor, purpura, hepatomegaly, splenomegaly, and lymphadenopathy. These results were in agreement with Yahya et al. 2013^21^ who found no significant differences between positive and negative BAALC gene expression regarding clinical parameters of patients at the time of diagnosis.

In this work, there were no significant differences between positive and negative BAALC gene expression regarding WBCs and platelets counts, hemoglobin, and LDH levels, and blast cell counts in the peripheral blood and bone marrow. This is in agreement with Elsharnouby et al. 2010^23^ who found no significant differences between positive and negative BAALC gene expression regarding WBCs and platelets counts, hemoglobin, and LDH levels, blast cell counts, both in the peripheral blood and bone marrow. But not in agreement with Baldus et al. 2003^24^ who found the association between positive BAALC gene expression and significantly higher WBCs and blast cell counts in the peripheral blood and bone marrow.

Variation between the results of this study and the previous studies may be explained by different age and number of studied patients, different localities, different presentations of leukemia, different duration of studies and different duration of follow-up.

In the current study, there was a significant association between BAALC gene expression and certain FAB subtypes with predominant positive BAALC gene expression in M1 and M2 and predominant negative BAALC gene expression in M4. These data are in agreement with Bienz et al 2005^26^ and Elsharnouby et al 2010 ^(23)^ who both found high BAALC expression more frequently in M1, M2 and less in M4, M5, and Yanaihara et al 2006^25^ who stated that high BAALC gene expression is more often present in M1.

In our study there were statistically significant differences in disease outcome between positive and negative BAALC gene expression groups with higher rate of relapse and death and lower rate of complete remission and disease free survival in positive BAALC gene expression group compared with negative BAALC gene expression group. This is in agreement with Nibourel et al 2010^27^ who stated that BAALC gene expression was found to be an independent negative prognostic factor in CN-AML, Yahya et al 2013^21^ who found that high BAALC expression had significantly lower incidence of CR, higher mortality rate, significantly shorter DFS, and inferior overall survival, Weber et al 2014^28^ who revealed an independent adverse prognostic impact of high BAALC expression on overall survival and event-free survival and Damiani et al 2013^22^ who found that overexpression of BAALC gene confers poor prognosis in cytogenetically normal AML patients, with negative impact on CR achievement, overall survival but have no influence on relapse probability.

## Conclusion and Recommendation

BAALC expression is an important bad prognostic factor in AML patients with normal karyotype, and therefore we recommend its incorporation into novel risk-adapted therapeutic strategies to improve the currently disappointing cure rate of patients with AML.

## Figures and Tables

**Table 1:** Comparison between patients with positive and negative BAALC gene expression regarding clinical and laboratory data.

Parameters		Positive BAALC gene expression group (n=24)		Negative BAALC gene expression group (n=16)		t test or X^2^	P- value

		N	%	N	%		

**Sex**	Males	14	35%	10	25%	0.260	0.610

	Females	10	25%	6	15%		

**Age (years)**	Mean ± SD	8.35±2.63		7.74±3.23		0.536	0.336

**Pallor**	Present	20	83.3%	13	81.25%	0.20	0.654

**Purpura**	Present	22	91.6%	14	87.5%	2.53	0.112

**Lymphadenopathy**	Present	8	33.33%	6	37.5%	0.361	0.547

**Hepatomegaly**	Present	16	66.66%	11	68.75%	0.095	0.758

**Splenomegaly**	Present	19	79.16%	13	81.25	0.49	0.481

**Hb (gm/dl)**	Mean ±SD	8.13±1.57		7.86±2.37		0.96	0. 632

**Platelets (×10^3^/mm^3^)**	Mean ±SD	69.86±33.15		77.13±48.07		1.85	0. 712

**TLC (×10^3^/mm^3^)**	Mean ±SD	75.51±51.31		76.44±46.67		0.93	0. 845

**Blast cells (%) in peripheral blood**	Mean ±SD	63 ±20.05		57.35±28.48		0.833	0.416

**BM blast cells (%)**	Mean ±SD	78.21±9.83		75.39±21.79		0.924	0.096

**LDH (U/L)**	Mean ±SD	1312.73± 695.63		1299.57±315.47		0.86	0.082

**FAB classification**							
**M1 (11 cases)**		9	37.5%	2	12.5%	3.510	0.04*
**M2 (17 cases)**		13	54.16%	4	25%	4.331	0.02*
**M4 (12 cases)**		3	12.5%	9	56.25%	3.359	0.031*

* Significant (p<0.05).

SD=Standard deviation. TLC= Total leucocytic count. BM=bone marrow. LDH=lactate dehydrogenase.

**Table 2:** Outcome of studied patients in relation to BAALC gene expression.

BAALC gene expression		Patients	outcome ( No=40)	
		Death (11 cases)	Remission (22 cases)	Relapse (7 cases)
**Positive BAALC gene expression group (No=24) (60%)**		8 (20)	10 (25)	6 (15)
**Negative BAALC expression group (No=16) (40%)**		3 (7.5)	12 (30)	1 (2.5)
**Total Number = 40 (100%)**		11 (27.5)	22 (55)	7 (17.5)
**Chi-square**	X^2^	5.421		
	P-value	0.017*		

* Significant.

**Table 3:** Log Rank test of overall and disease-free survival.

	Overall survival			Log Rank	
	Median	SE	CI 95%	test value	P-value
**Negative**	22.110	0.970	(20.21–24.01 )	1.750	0.185
**Positive**	16.840	2.070	(12.79–20.90)		
	**Disease free survival**			**Log Rank**	
	**Median**	**SE**	**CI 95%**	**test value**	**P-value**
**Negative**	21.630	1.010	( 19.650–23.610 )	5.450	0.020*
**Positive**	6.030	0.880	( 4.310–7.750 )		

* Significant.

SE=Standard error. CI= Confidence Interval.
